# Navigating the nexus: synergistic challenges in diabetes, hypertension, and renal medicine

**DOI:** 10.1080/0886022X.2026.2709819

**Published:** 2026-08-11

**Authors:** Omar Ahmed, Hatem Ali, Shafi Malik

**Affiliations:** ^a^ Medical Department, Salford Royal Hospital, Greater Manchester, UK; ^b^ Renal Department, University Hospital of Wales, Cardiff, UK; ^c^ Renal Department, University Hospital of Birmingham, Birmingham, UK

Chronic kidney disease (CKD) has become one of the defining global health challenges of the modern era, driven largely by the parallel epidemics of diabetes mellitus and hypertension. Together, these conditions account for the majority of progressive kidney disease worldwide and are increasingly recognized not as isolated diagnoses, but as tightly interconnected processes that amplify vascular injury, metabolic stress, inflammation, and nephron loss. Diabetes accelerates glomerular hyperfiltration, oxidative stress, and structural damage, while hypertension compounds endothelial dysfunction, proteinuria, and progressive fibrosis. Their coexistence creates a clinical nexus in which renal decline is often faster, cardiovascular risk higher, and therapeutic complexity greater than with either condition alone.

This article collection in *Renal Failure* arrives at an important time. Despite substantial advances in renoprotective therapies—including renin–angiotensin system blockade, mineralocorticoid receptor antagonism, and sodium-glucose cotransporter-2 (SGLT2) inhibitors—the burden of CKD attributable to diabetes and hypertension continues to rise. Clinicians increasingly face questions that extend beyond simple blood pressure or glycaemic thresholds: how can risk be detected earlier, outcomes predicted more accurately, metabolic instability reduced, and molecular pathways interrupted before irreversible fibrosis develops? The studies assembled in this collection address these challenges from complementary perspectives, spanning acute clinical biomarkers, dialysis outcomes, nutritional epidemiology, cellular therapeutics, and epigenetic regulation.

A particularly notable contribution is the study by Lin and colleagues examining the stress hyperglycemia ratio (SHR) as a predictor of acute kidney injury (AKI) and its outcomes in critically ill patients [1]. Traditionally, clinicians have relied on absolute glucose values when assessing stress responses in hospitalized patients. Yet glucose concentration alone may inadequately distinguish chronic hyperglycemia from acute metabolic stress. The SHR, by adjusting admission glucose relative to background glycaemic status, offers a more nuanced index of physiological disturbance. This is clinically relevant because AKI in critical illness often develops within a broader syndrome of haemodynamic compromise, inflammation, and metabolic dysregulation. If validated prospectively, SHR could become a practical tool to identify patients at heightened renal risk earlier in the course of illness.

More broadly, this study reflects an important shift in nephrology: movement from static biochemical thresholds toward context-sensitive biomarkers. Creatinine, glucose, and blood pressure values each gain meaning when interpreted relative to baseline physiology and acute trajectory. Future work should determine whether dynamic metabolic markers such as SHR can be integrated into real-time risk prediction models, prompting earlier fluid optimization, medication review, or nephroprotective interventions.

If acute hyperglycemia represents one side of metabolic stress, hypoglycemia represents the other. Shi and colleagues provide compelling evidence that frequent hypoglycemic episodes during hemodialysis are associated with increased mortality among patients with end-stage renal disease [2]. This finding deserves particular attention. Dialysis populations often have altered insulin clearance, impaired counter-regulatory responses, variable nutritional intake, and rapid shifts in glucose handling during treatment sessions. As a result, glycaemic targets extrapolated from the general diabetes population may not always be appropriate in dialysis units.

The clinical implications are substantial. Hypoglycemia may act not only as a marker of frailty and advanced disease, but also as a direct mediator of adverse outcomes through arrhythmia, sympathetic activation, falls, cognitive injury, and cardiovascular stress. These data support a more individualized approach to glucose management in dialysis patients—one that prioritizes safety and stability rather than aggressive normalization. Continuous glucose monitoring, dialysis-specific medication adjustments, and modified dialysate strategies may become increasingly relevant. Importantly, the study invites a broader reappraisal of metabolic variability as a therapeutic target. In some patients, avoiding extremes may matter as much as lowering averages.

The collection then moves upstream from clinical events to disease causation and prevention. Zhang and colleagues use Mendelian randomization to explore the relationship between dietary habits and diabetic kidney disease (DKD) [3]. Nutritional research has historically been challenged by confounding, recall bias, and reverse causation. By using inherited genetic variants as proxies for behavioral exposures, Mendelian randomization offers an innovative framework to strengthen causal inference.

Such work is timely. Diet remains one of the most modifiable yet underused tools in CKD prevention. Excess sodium intake, obesity-promoting dietary patterns, refined carbohydrates, and low fiber consumption all intersect with hypertension, insulin resistance, and renal injury. Although genetic predisposition to dietary behavior does not fully replicate real-world nutrition, studies of this kind help identify which exposures warrant more rigorous interventional testing. They also reinforce an important message: prevention of diabetic kidney disease begins long before albuminuria appears. The future of nephrology will depend not only on better dialysis and transplantation, but also on earlier metabolic prevention at population scale.

At the therapeutic frontier, Tian and colleagues provide one of the most mechanistically intriguing studies in the collection. Their work suggests that dapagliflozin improves diabetic kidney disease by inhibiting ferroptosis through increased β-hydroxybutyrate production [4]. This is an important conceptual advance. SGLT2 inhibitors are now established as cornerstone therapies in diabetic and non-diabetic CKD, yet their full mechanisms of benefit remain incompletely understood. Initially framed largely in haemodynamic terms—reducing intraglomerular pressure and hyperfiltration—it is increasingly apparent that these agents also influence inflammation, mitochondrial energetics, oxidative stress, and cellular survival pathways.

Ferroptosis, an iron-dependent form of regulated cell death driven by lipid peroxidation, has emerged as a candidate pathway in renal injury. Linking dapagliflozin-mediated ketosis to suppression of ferroptosis broadens our understanding of how metabolic therapies may confer structural kidney protection. It raises provocative future questions. Could β-hydroxybutyrate itself become a therapeutic mediator? Might nutritional ketosis or targeted metabolic substrates replicate some benefits of SGLT2 inhibition in selected patients? Could biomarkers of ferroptotic activity help personalize treatment selection? While translational caution is necessary, studies such as this illustrate the growing convergence of metabolism and molecular nephrology.

Completing the collection is the review by Hu and colleagues on histone methylation in kidney disease [5]. This topic addresses one of the most persistent frustrations in renal medicine: why do some patients continue to progress despite apparently optimal control of blood pressure, glycemia, and conventional risk factors? Increasingly, the answer may lie in maladaptive cellular memory. Prior metabolic injury can leave durable epigenetic marks that sustain inflammatory and fibrotic pathways even after the initiating insult has improved.

Histone methylation is among the most promising of these mechanisms. By regulating chromatin accessibility and gene transcription, histone modifications may influence podocyte injury, tubular inflammation, mesangial expansion, and fibroblast activation. If validated therapeutically, epigenetic targeting could represent a new treatment paradigm—one aimed not merely at controlling external risk factors, but at reprogramming the kidney’s internal response to chronic injury. Although much remains experimental, the concept is compelling: future nephrology may involve treating biological memory as much as treating blood pressure or glucose.

Taken together, the papers in this collection reveal a common theme. Kidney disease in diabetes and hypertension is not solely the consequence of elevated glucose or elevated pressure. Rather, it is the downstream result of repeated metabolic insults, haemodynamic strain, inflammatory signaling, oxidative injury, altered cellular energetics, and maladaptive repair. These studies collectively move the field beyond simplistic metrics toward a multidimensional understanding of renal vulnerability.

Several practical lessons emerge. First, risk detection must become more dynamic, incorporating biomarkers that reflect stress and trajectory rather than isolated values alone. Second, metabolic stability deserves greater emphasis, particularly in vulnerable populations such as dialysis patients. Third, prevention strategies—including nutrition and lifestyle intervention—remain essential and should be pursued with the same seriousness as pharmacotherapy. Fourth, the therapeutic future of nephrology lies increasingly in pathway-based medicine, where agents such as SGLT2 inhibitors and potential epigenetic modulators act on shared mechanisms of injury.

The challenge ahead is integration. Clinicians need tools that combine metabolic, haemodynamic, and molecular risk into actionable bedside strategies. Researchers need trials that move from association to intervention. Health systems need models that detect CKD earlier in populations burdened by diabetes, hypertension, and socioeconomic disadvantage. This collection contributes meaningfully to all three aims.

In that sense, the nexus of diabetes, hypertension, and kidney disease should not be viewed solely as a crisis. It is also an opportunity: to redesign renal care around earlier prediction, smarter prevention, and biologically targeted protection of kidney function. We hope this collection stimulates continued work toward that goal.

## Data Availability

No new data were generated or analyzed in this editorial. Data sharing is therefore not applicable to this article.
